# Assessing Transportation Barriers to Maternal Care for Black Women in Los Angeles County

**DOI:** 10.3390/ijerph22091429

**Published:** 2025-09-13

**Authors:** Rebecca O. Usigbe, Zanobia R. Ibrahim-Watkins, Astrid Williams, Sylvie Wilson, Zoe Cunliffe, Gabrielle Brown, Tianna Shaw-Wakeman, Regan F. Patterson

**Affiliations:** 1 Institute of Society and Genetics, University of California, Los Angeles, CA 90095, USA; rebeccaus128@ucla.edu; 2 Department of Civil and Environmental Engineering, University of California, Los Angeles, CA 90095, USA; zibrahim@ucla.edu; 3 California Black Health Network, Sacramento, CA 95814, USA; awilliams@cablackhealthnetwork.org (A.W.); swilson@cablackhealthnetwork.org (S.W.); 4 Black Women for Wellness, Los Angeles, CA 90008, USA; zoe@bwwla.com (Z.C.); gabrielle@bwwla.com (G.B.); tianna@bwwla.com (T.S.-W.)

**Keywords:** transportation access, public health, black maternal health, racial health disparities, environmental justice

## Abstract

The United States ranks among the worst high-income countries for maternal health outcomes, with Black women experiencing disproportionately high and alarming rates of maternal mortality and morbidity. In Los Angeles County, Black women are four times more likely to die from pregnancy-related causes than women of other racial and ethnic groups. These disparities may partially be attributed to social determinants of health, including transportation access. Lack of transportation can hinder access to healthcare, with significant consequences for reproductive health. This study investigates how transportation barriers affect Black birthing people’s access to maternal healthcare in Los Angeles. In partnership with Black Women for Wellness, we conducted a descriptive, observational study using an online survey completed by 235 respondents, all of whom self-identified as women. Findings reveal that Black women in Los Angeles face substantial transportation barriers when seeking maternal healthcare, including limited public transportation, lack of personal vehicles, and challenges in securing rides. Many participants reported that these issues caused delayed or missed prenatal appointments. These results underscore the urgent need for policy interventions and systems-level solutions to improve transportation access. Addressing these barriers is essential for reducing maternal health disparities and improving outcomes for Black women.

## 1. Introduction

### 1.1. Maternal Health Crisis in the United States

The maternal mortality rate refers to the number of maternal deaths per 100,000 live births among those who are pregnant or within 42 days of termination of pregnancy [1]. In the United States, the maternal mortality rate has been steadily increasing, despite advances in modern medicine [1]. While the average maternal mortality rate in high-income countries is 12 per 100,000 live births [2], the United States had a maternal mortality rate of 18.6 per 100,000 live births in 2023 [3].

When the maternal morbidity and mortality data are stratified by racial group, the reality becomes even more grim. A 2024 study revealed that Black individuals were 1.9 times more likely than white women to experience intrapartum maternal morbidity, but the rate spikes to 2.5 times more likely than their white counterparts during the postpartum period [4]. In 2023, the maternal mortality rate among white women was 14.5 deaths per 100,000 live births, while Black women faced a maternal mortality rate of 50.3, more than three times the rate of their white counterparts [3].

In Los Angeles County specifically, these disparities are even more pronounced. Black birthing people face a maternal mortality rate four times higher than other racial groups [5]. They also experience a higher risk of cesarean sections and preterm births, while Black infants are three times more likely to die than white and Asian infants [5,6,7,8]. Although cesarean sections are necessary in some cases, they are associated with increased risks of health complications after birth [9].

### 1.2. Transportation Access as a Social Determinant of Health

Public health officials turn to the social determinants of health to explain the persistence of health disparities. Social determinants of health are non-medical factors that influence one’s health outcomes, such as income, education, food insecurity, and access to transportation [10].

Several studies explore inadequate transportation as a barrier to healthcare access in the United States. For instance, the National Center for Health Statistics found that, in 2022, 5.7% of adults did not have reliable transportation to conduct daily living tasks such as healthcare appointments. While the study did not provide statistics specifically for Black women, both identities face higher rates than the average, with 6.1% of women with no reliable transportation and 9.2% of Black individuals without reliable transportation [11]. Similarly, a North Carolina study found that one-third of people who had six or more medical appointments between 2020 and 2021 experienced transportation-related challenges. The most frequent types of transportation barriers mentioned were the lack of access to a driver or car and the cost of travel [12]. While these studies illuminate the relationship between transportation and healthcare broadly, the specific connection between transportation access and maternal healthcare, particularly for Black birthing people, remains largely unexplored.

Barriers to transportation can manifest across multiple levels of the Socio-Ecological Model, which provides a framework for understanding how health behaviors and outcomes are shaped by individual, interpersonal, community, and societal factors. At the individual level, a pregnant person may not have access to a vehicle for their prenatal appointments. Interpersonally, they may have to rely on others in their social network in order to access transportation to a healthcare provider, which may not always be reliable. At the community level, limited public transit options or the absence of nearby maternity wards can make maternal healthcare inaccessible. Lastly, societal factors, such as underinvestment in low-income areas and structural racism may exacerbate transportation inequities. For instance, the median household income in communities where maternity wards closed was $12,000 less than in communities where they remained open [13]. The Socio-Ecological Model thus provides a valuable theoretical framework for examining how transportation barriers operate at multiple, inter-related levels in accessing maternal healthcare.

### 1.3. Closures of Maternal Health Wards

Transportation barriers are further intensified by increased travel times due to maternity ward closures in California. The number of hospitals with labor and delivery services in California has diminished from 250 to 214 since 2012, with three of the hospitals completely closing [14]. Between 2012 and 2019, 19 hospitals in California closed their labor and delivery services, followed by 16 additional closures from 2020 to 2022, 11 more in 2023 [14], and another 7 in 2024 [15].

In Los Angeles County specifically, 15 hospitals closed their labor and delivery services. As a result, only 53 hospitals in Los Angeles County offer maternal care as of 2023 [14]. For example, in 2024, USC Verdugo Hills Hospital closed its 18-bed Labor and Delivery unit and directed patients to nearby alternatives such as USC Arcadia Hospital [16]. Though this facility is just 4.7 miles away, travel time can reach an hour on public transportation during peak traffic time. Similarly, the nearest Federally Qualified Health Center (FQHC), Glendale’s Comprehensive Community Health Center, is 7.5 miles away and can require up to 50 min of travel due to traffic volumes in Los Angeles County, which is 1.5 times higher than the California average and nearly 7 times higher than the national average [17]. While FQHCs provide prenatal and preventive services, they do not offer inpatient labor and delivery services, leaving significant gaps in coverage.

These closures increase both the distance and time required to access maternal care, making it more difficult for patients with limited transportation access. Long travel times on public transportation can make it increasingly difficult for expectant mothers to access care, and these extended journeys may also contribute to longer wait times upon arrival. These developments highlight critical community-level and societal-level barriers that restrict maternal healthcare access for underserved populations, particularly Black birthing people in Los Angeles County.

### 1.4. Purpose of the Study

This study aims to understand the role of transportation barriers in access to maternal healthcare for Black birthing people in Los Angeles County. Addressing these barriers is essential for closing racial disparities in maternal health outcomes and reducing maternal morbidity and mortality rates.

Our analysis is exploratory, and while we do not pose a formal hypothesis, we seek to illuminate key access issues and inform future interventions and policy efforts.

## 2. Materials and Methods

### 2.1. Study Design and Population

To assess Black birthing people’s access to transportation and access to healthcare, a descriptive, observational study was conducted using online surveys. The study received a Category 2 exemption by the Institutional Review Board at the University of California, Los Angeles, as all participant data remained anonymous.

We collaborated with Black Women for Wellness, a woman-centered, grassroots, community-based organization that advocates for the health and reproductive justice of Black women, their families, and future generations. Together, we co-created an online survey designed to explore transportation challenges and maternal healthcare experiences.

Participants were recruited for the study through Black Women for Wellness. They were eligible to participate if they (1) identified as African American or Black; (2) had given birth between January 2023 and April 2024, or were pregnant at the time of survey completion; (3) lived in Los Angeles County; and (4) were 18 years old or older. This study utilized convenience sampling, with outreach conducted via Instagram, email lists, and phone banking. Participation was entirely voluntary and anonymous.

### 2.2. Survey Instrument

The survey instrument was structured using the Socio-Ecological Model as a guiding framework to examine transportation barriers to maternal healthcare across four levels: individual, interpersonal, community, and societal. At the individual level, the survey assessed factors such as personal car access and travel costs. The interpersonal level included questions about reliance on others for transportation and the availability of social support networks. At the community level, the survey explored issues like proximity to hospitals, availability of public transportation, and travel times. Finally, societal factors were examined through questions related to neighborhood infrastructure and the safety of transportation options.

The final Qualtrics survey included 36 questions. Before beginning the survey, participants were presented with a research information sheet outlining the study’s purpose, procedures, and participant rights. Informed consent was obtained by requiring participants to confirm they had read and agreed to the information provided before proceeding.

The survey was structured in four main sections. Initial screening questions assessed eligibility based on racial identity, age, pregnancy or postpartum status, and residency in Los Angeles County, which was verified by zip code. No personally identifiable information was collected in order to preserve participant anonymity.

The survey then collected sociodemographic data including gender identity, relationship status, education, employment, household size, income, insurance status, and healthcare out-of-pocket cost.

Subsequent questions focused on transportation and healthcare access, including questions on distance to their maternal healthcare provider, personal access to a vehicle, primary mode of transport, travel time, timeliness to appointments, and experiences with transportation barriers. These questions were directly informed by the Socio-Ecological Model framework.

Finally, participants were asked to rank potential government interventions to improve transportation access and were invited to share open-ended responses reflecting on their overall experiences accessing maternal healthcare.

The survey included a variety of question formats, such as multiple-choice, open-ended, yes/no responses, and Likert scale. To protect anonymity, no identifying information was collected.

Of the 1528 people who took the survey, 235 surveys were included as valid in our study. Selection criteria included the completion of the entirety of the survey, responses to open-ended questions, and participant engagement of at least ten minutes to complete the survey. The time requirement was established in order to ensure participants were reading and understanding the questions asked of them. Surveys were excluded if they did not meet eligibility criteria (*n* = 68), submitted incomplete open-ended responses, or completed the survey in under 10 min (*n* = 1225). Because only fully completed surveys were included, there were no missing data in the final dataset. Eligible survey respondents received a $25 gift card as compensation for their participation.

### 2.3. Data Analysis

All quantitative data were analyzed using descriptive statistics to summarize participant responses. Analyses were conducted using Google Sheets. Given the exploratory nature of the study and non-random sampling approach, no inferential statistical tests were performed. Open-ended responses were reviewed to identify recurring themes and were used to complement the quantitative findings.

## 3. Results

### 3.1. Demographics

Although this study aimed to examine transportation barriers for birthing people, all 235 of the survey respondents identified as women. Therefore, findings will be discussed in terms of women’s experiences, while recognizing that not all birthing people identify as women.

The majority of participants were between the ages of 25–34, comprising 86% of the sample. A total of 7% were aged 18 to 24, and another 7% were between 35 and 44 (Table 1). Most women (91%) had at least some college experience, and 82% worked full-time at the time of participation. Regarding household income, 69% reported earning between $25,000 and $49,999 annually.

Of the total participants, 160 had given birth in 2023 or 2024 (Table 1). Among women who were currently pregnant during the time of the survey, 29 were in their third trimester, 33 were in their second trimester, and 13 were in their first trimester. A total of 90% of respondents had one or two children, which included the child they were currently pregnant with, while the remaining 10% had three to four children.

Private health insurance provided by employers covered most participants (91%), yet 80% reported paying out of pocket for healthcare visits, with costs ranging from $30 to $1000. Seven percent received Medi-Cal or Medicaid benefits. Of the participants that have a primary care provider, 156 went to a hospital for care, 48 went to a physician/doctor and 24 went to a clinic. One participant indicated that they received care from a doula.

### 3.2. Transportation Access

**Figure 1:**
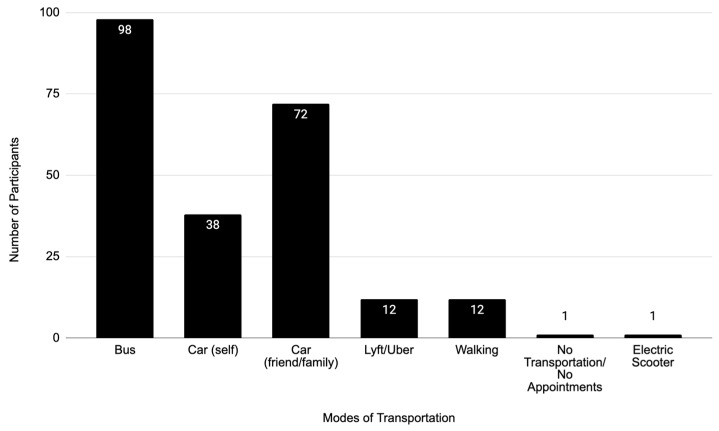
Primary mode of transportation to maternal health appointments.

A majority of new or expectant mothers, 67%, did not own or have access to a car. The primary transport mode reported by respondents was the bus (Figure 1). However, most participants (83%) used multiple modes of transportation, with the most common being car (47%), bus (42%), rideshare service (5%), and walking (5%).

**Figure 2:**
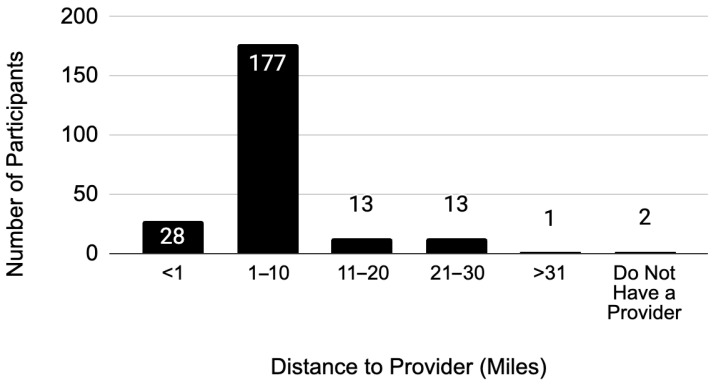
Distance to primary maternal health provider.

Most respondents (75%) spent between $1 and $10 on one-way transportation costs. For the same proportion, the distance to their primary maternal health provider ranged from 1 to 10 miles (Figure 2). The average one-way travel time for respondents varied widely, from 0 to 79 min, with the most frequently reported travel time being 20 to 39 min (38%).

### 3.3. Effect of Transportation on Access to Maternal Healthcare

During pregnancy, 81% of participants experienced changes in their transportation access. Among those who reported changes, 13% noted that the change was due to transportation becoming more expensive. Other factors influencing these changes included driver availability, fluctuations in appointments times, and availability of public transportation or rideshare services.

Another major factor driving changes in transportation mode was the decline in comfort with certain modes of transportation as their pregnancy progressed. For example, many women expressed feeling unsafe taking public transportation, with some even recounting experiences of receiving “disgusted looks” while on the bus.

Transportation difficulties were a major barrier to accessing care. Nearly three quarters (73.6%) identified transportation issues as the primary reason for late, missed or rescheduled appointments. While some participants did not view transportation as the primary issue, 91% of respondents acknowledged they have experienced problems with their appointments due to transportation, and 89% explicitly identified transportation as a barrier that limits their ability to access maternal healthcare. The most commonly reported transportation barrier was the lack of public transit, which affected 69% of participants (Table 2).

### 3.4. Safety

Safety concerns while traveling during pregnancy were reported by a meaningful number of study participants. Specifically, 14% expressed feeling unsafe using their specific transport mode (e.g., bus, train, or rideshare). Additionally, 6% felt unsafe in either their own neighborhood or the neighborhood of their primary care provider due to concerns about stalking, crime, traffic, or the risk of falling on sidewalks. Another 4% mentioned other safety concerns. For instance, one mother recounted being scrutinized and judged while waiting for the bus, and another shared that she was “handled recklessly” during transportation. Multiple participants also reported feeling unsafe due to the distance to their primary care provider or the lack of a direct bus route to their appointment location.

## 4. Discussion

This study highlights that many Black women in Los Angeles County face substantial transportation barriers when accessing maternal healthcare. While findings cannot be generalized beyond the sample, the results point to structural inequities that disproportionately affect Black communities and contribute to racial disparities in maternal health outcomes.

One notable insight was that some women in the study reported feeling uncomfortable and unsafe taking public transportation, which is consistent with broader research showing that women experience high rates of sexual harassment and assault while using public transit. These risks are often magnified for Black women due to the intersection of racism and sexism, which increases their vulnerability to violence [18]. These safety concerns are not just personal experiences but systemic public health issues that must be addressed through policy and urban planning. California Senate Bill (SB) 1161, signed into law in 2022, mandates that large transit agencies examine rider safety and street harassment within their systems [19]. However, once these safety concerns are exposed, they must be followed by concrete policies to meaningfully improve the safety of public transportation for Black women and other vulnerable riders.

Geographic access emerged as a key barrier in our survey. To contextualize our findings, we examined hospital and public transportation data for the three zip codes with the highest participant representation: 90003, 90044, and 90043. In 90003, there were three hospitals within a five-mile radius that accepted Medicare and provided inpatient labor and delivery care. In the 90043 and 90044 zip codes, there are two and three Medicare-certified hospitals providing these services, respectively [20]. While this distance may seem manageable, participants’ responses align with data showing that travel times can reach up to an hour during peak traffic times due to congestion and transit delays. Furthermore, Metro buses in Los Angeles arrive over five minutes late on average 22% of the time [21], further exacerbating delays. These combined factors, limited hospital proximity, unreliable transit, and heavy traffic, can help explain the transportation barriers participants described, linking their lived experience to broader spatial and infrastructural challenges. Collaboration between maternal health providers and public transportation agencies may help address these gaps by coordinating services, improving transit routes to clinics, and prioritizing safety and reliability for new or expectant riders.

While identifying these barriers is critical, it is equally important to find suitable solutions that will benefit the community. Participants were given the opportunity to voice some solutions they felt would be effective. They offered several ideas, including closer healthcare institutions, access to telemedicine, an increase in mobile clinics, and more affordable transportation options.

Telemedicine was ranked as the most helpful solution by participants, and it could be pivotal in delivering healthcare to the 67% of women in the study who did not have access to a car. Virtual visits allow patients to attend appointments from the comfort of their homes, reducing travel burdens. However, if the birthing person does not have access to technology, there is still a possibility that they cannot access the healthcare that they need. It is also important for birthing people to be able to see their provider in person, which is not feasible with telemedicine. Mobile clinics provide a solution that addresses some of the shortcomings of telemedicine. They were the second most preferred solution. These clinics can be stationed in areas of maternal healthcare deserts and give patients the opportunity to receive routine checkups. As previously mentioned, many maternal health wards in California are closing their doors. With fewer healthcare institutions offering obstetric services, patients will have to travel farther distances in order to get to their appointments. These extended commutes can require multiple modes of transportation, compounding logistical challenges and heightening safety concerns, particularly for those who rely on public transit. By bringing services directly into underserved neighborhoods, mobile clinics can bridge some of the physical and financial gaps in access and may help mitigate the risks and burdens associated with long, multi-modal journeys to get to their distant appointments.

Affordability was another major theme. More affordable transportation was recommended as a solution by many participants. California has established policies that could help alleviate this issue. For instance, California’s Medicaid healthcare program for low-income residents, Medi-Cal, offers transportation services. Individuals who are pregnant or within one year postpartum can access Non-Medical Transportation (NMT) and Non-Emergency Medical Transportation (NEMT) [22]. NMT offers public and private transportation services, arranging rides to medical appointments for expecting and new mothers who lack other viable transportation options. This service is also available to individuals who fall outside that group if they have full-scope Medi-Cal coverage. NEMT includes ambulances, wheelchair vans, or litter vans for those who are unable to use public or private transportation.

In Los Angeles specifically, residents with Medi-Cal are eligible for L.A. Care, a health plan that offers free transport to appointments through a service called, “Call the Car” [23]. Los Angeles County Health Services also offers free Uber and Lyft rides to and from appointments for individuals who are not eligible for other free transportation services [23].

Given some women in the study reported feeling unsafe using rideshare services, it is essential to assess the safety and reliability of these transportation options. While Medi-Cal’s NMT service is available to give free transportation to appointments for eligible birthing people, many are unaware that such programs exist. Increasing awareness of existing transportation benefits, ensuring medical benefits receive appropriate exposure, and expanding service options are critical steps in order to alleviate transportation barriers to maternal healthcare.

Although more affordable public transportation was one proposed solution, it is important to note that bus passes and rideshare vouchers were ranked lowest among solutions. This may be due to the previously mentioned safety concerns amongst participants. This underscores the need to prioritize safety in any transportation intervention aimed at advancing maternal health equity, particularly for expectant Black mothers who face heightened risks.

### Limitations

While the survey revealed important insights, several limitations should be considered when interpreting the findings. Although this study aimed to focus on the experiences of Black birthing people, all respondents self-identified as women. Other gender identities that access maternal healthcare may have different experiences that were not captured in this sample. Future research should intentionally seek to recruit and include a more gender-diverse population.

While this study focused on how race may contribute to transportation barriers, other factors, such as socioeconomic status and insurance coverage, may play a role. The study relied on self-reported data, and there remains a possibility that some participants were not fully truthful about their identities or experiences, introducing the potential for sampling bias. Additionally, the survey was not pilot-tested or formally validated through psychometric analysis. However, it was co-developed with community partners to reflect real-world experience and local context. This approach prioritized cultural relevance and community trust over broad generalizability.

Participants were recruited through the community-based organization, Black Women for Wellness. While this partnership enabled strong community engagement, it may have introduced selection bias, particularly if individuals who experience transportation barriers were more motivated to respond. Furthermore, a total of 1225 responses were excluded due to incomplete answers or rapid survey completion. This may have introduced non-response bias, as individuals with limited time, such as busy or working mothers, may not have had the time to complete the survey and would not have been represented in the data.

Finally, the study employed a descriptive, exploratory design and utilized convenience sampling rather than a stratified or randomized sampling approach. Due to the non-random sample and the study’s exploratory nature, inferential statistical analyses were not conducted. As such, we cannot draw conclusions about statistical associations or causal relationships between transportation barriers and participant characteristics, such as race, income, insurance status, or transportation mode. Future research should use representative sampling and multivariate analysis to explore how specific factors influence access to maternal healthcare.

## 5. Conclusions

This study found that many Black birthing people in our sample experienced substantial transportation barriers when attempting to access maternal healthcare in Los Angeles County. The main transportation barriers discovered were lack of public transit, lack of a car, and difficulty finding someone to drive. These transportation obstacles can delay or prevent timely care during pregnancy, with serious implications for maternal health.

These findings suggest a need for policy changes to enhance birthing peoples’ experiences with accessing healthcare. While some existing programs, such as NEMT and rideshare initiatives, aim to address these gaps, barriers remain due to lack of awareness and feelings of unsafety. A more in-depth exploration on developing and implementing policies and practices that address transportation access is still required. Additional research is needed to examine how increased awareness, improved transit safety, and changes to transportation policies could improve maternal health outcomes.

Participants also proposed practical solutions, including expanded telemedicine, more mobile clinics, and greater affordability and safety in transit systems. Listening sessions should be conducted with Black birthing people to better understand transportation barriers and co-create solutions. This is a matter of equity, and in many cases, it is truly a matter of life and death. Transportation should never be the barrier that prevents someone receiving life-saving care.

## Figures and Tables

**Table 1:** Demographic and other characteristics of participants.

Characteristic	Category	Count (Percent)
Age	18–24	17 (7.2%)
	25–34	202 (86.0%)
	35–44	16 (6.8%)
	>45	0
Education	Less than High School	2 (0.8%)
	Some High School	3 (1.2%)
	High School or Equivalent	14 (6.0%)
	Trade/Technical School	6 (2.6%)
	Associate’s Degree	22 (9.4%)
	Some College	163 (69.4%)
	Bachelor’s Degree	23 (9.8%)
	Master’s/Professional Degree	2 (0.8%)
Annual Household Income	<USD 25,000	8 (3.4%)
	USD 25,000–49,999	163 (69.4%)
	USD 50,000–74,999	20 (8.5%)
	USD 75,000–99,000	24 (10.2%)
	USD 100,000–124,999	11 (4.7%)
	USD 125,000–149,999	3 (1.3%)
	USD 150,000–174,999	4 (1.7%)
	USD 175,000–199,999	1 (0.4%)
	Prefer Not to Disclose	1 (0.4%)
Pregnancy Status	First Trimester	13 (5.5%)
	Second Trimester	33 (14.1%)
	Third Trimester	29 (12.3%)
	Gave Birth in 2023/2024	160 (68.1%)
Medical Insurance	Private Insurance—Employer-Provided	196 (83.5%)
	Private Insurance—Individual Health Plan	17 (7.2%)
	Medi-Cal Access Program or Medicaid	17 (7.2%)
	Not Currently Insured	4 (1.7%)
	Private Insurance—College- or University-Provided	1 (0.4%)
Primary Care Provider	Hospital	156 (66.4%)
	Physician	48 (20.4%)
	Clinic	24 (10.2%)
	No Primary Care Provider	5 (2.2%)
	Doula *	1 (0.4%)
	Prefer Not to Disclose	1 (0.4%)

* Doulas do not provide prenatal care services, but provide support for birthing people.

**Table 2:** Transportation barriers to accessing maternal healthcare.

Transportation Barriers (Select All That Apply)	Count	Percentage
Lack of Public Transit	162	69%
Lack of a Car	105	45%
Finding Someone to Drive	77	33%
Distance to Care	56	24%
Cost of Transport	52	22%
Feel Unsafe in Transport More	33	14%
No Transportation Barriers Reported	13	6%

## Data Availability

The anonymized data presented in this study are available on request from the corresponding author. Access is restricted to ensure alignment with ethical standards for human subjects research.
